# Immune-Redox Biomarker Responses to Short- and Long-Term Exposure to Naturally Emitted Compounds from Korean Red Pine (*Pinus densiflora*) and Japanese Cypress (*Chamaecyparis obtusa*): In Vivo Study

**DOI:** 10.3390/toxics13080650

**Published:** 2025-07-31

**Authors:** Hui Ma, Jiyoon Yang, Chang-Deuk Eom, Johny Bajgai, Md. Habibur Rahman, Thu Thao Pham, Haiyang Zhang, Won-Joung Hwang, Seong Hoon Goh, Bomi Kim, Cheol-Su Kim, Keon-Ho Kim, Kyu-Jae Lee

**Affiliations:** 1 Department of Global Medical Science, Wonju College of Medicine, Yonsei University, Wonju 26426, Republic of Korea; mahui56@yonsei.ac.kr; 2 Department of Convergence Medicine, Wonju College of Medicine, Yonsei University, Wonju 26426, Republic of Korea; johnybajgai@yonsei.ac.kr (J.B.); globaldreamer1990@gmail.com (M.H.R.); phamthuthaoytcc@gmail.com (T.T.P.); zhanghyzrc1017@gmail.com (H.Z.); forget419@hanmail.net (S.H.G.); kimbomi9090@gmail.com (B.K.); cs-kim@yonsei.ac.kr (C.-S.K.); 3 Department of Orthopedic Surgery, People’s Hospital of Fengjie County, Chongqing 404600, China; 4 Division of Wood Industry, Department of Forest Products and Industry, National Institute of Forest Science, Seoul 02455, Republic of Korea; dldh89@korea.kr (J.Y.); willyeom@korea.kr (C.-D.E.); wonjoung@korea.kr (W.-J.H.); 5 Department of Laboratory Medicine, Wonju College of Medicine, Yonsei University, Wonju 26426, Republic of Korea

**Keywords:** volatile organic compounds (VOCs), wood exposure, oxidative stress, toxicity, inflammation

## Abstract

Volatile organic compounds (VOCs) are highly volatile chemicals in natural and anthropogenic environments, significantly affecting indoor air quality. Major sources of indoor VOCs include emissions from building materials, furnishings, and consumer products. Natural wood products release VOCs, including terpenes and aldehydes, which exert diverse health effects ranging from mild respiratory irritation to severe outcomes, such as formaldehyde-induced carcinogenicity. The temporal dynamics of VOC emissions were investigated, and the toxicological and physiological effects of the VOCs emitted by two types of natural wood, Korean Red Pine (*Pinus densiflora*) and Japanese Cypress (*Chamaecyparis obtusa*), were evaluated. Using female C57BL/6 mice as an animal model, the exposure setups included phytoncides, formaldehyde, and intact wood samples over short- and long-term durations. The exposure effects were assessed using oxidative stress markers, antioxidant enzyme activity, hepatic and renal biomarkers, and inflammatory cytokine profiles. Long-term exposure to Korean Red Pine and Japanese Cypress wood VOCs did not induce significant pathological changes. Japanese Cypress exhibited more distinct benefits, including enhanced oxidative stress mitigation, reduced systemic toxicity, and lower pro-inflammatory cytokine levels compared to the negative control group, attributable to its more favorable VOC emission profile. These findings highlight the potential health and environmental benefits of natural wood VOCs and offer valuable insights for optimizing timber use, improving indoor air quality, and informing public health policies.

## 1. Introduction

Volatile organic compounds (VOCs) are a diverse group of organic chemicals characterized by high volatility, enabling their rapid evaporation at ambient temperatures [1]. These compounds primarily consist of aromatic, alkane, and alkene moieties, which are widely detected in atmospheric environments [2]. VOCs are integral to various industrial applications, including paints, varnishes, adhesives, and cleaning agents, and they also occur naturally in vegetation and wood products [3]. The World Health Organization defines VOCs as organic compounds with boiling points ranging from 50–100 °C to 240–260 °C, encompassing semi-volatile organic compounds and very volatile organic compounds [4].

Indoor air pollution caused by VOCs is a major concern, especially in developed countries, owing to its adverse effects on public health [5]. Common indoor sources of VOCs include building materials, furniture, and consumer products, such as personal care items and air fresheners [6,7]. Among them, wood-based products are notable sources of VOCs. Natural wood emits VOCs such as terpenes and aldehydes, whereas engineered wood products release synthetic VOCs, including formaldehyde, through adhesives and finishes [8,9]. These emissions contribute to the deterioration of indoor air quality and are associated with health syndromes, such as sick building syndrome [4,10].

The health effects of VOC exposure vary depending on its concentration and duration. Low-level exposure can cause mild respiratory or ocular irritation, whereas chronic exposure to substances, such as formaldehyde, has been classified as carcinogenic by the International Agency for Research on Cancer [11,12]. However, certain VOCs emitted from natural wood, particularly terpenes such as α-pinene and β-pinene, have been demonstrated to have properties that provide potential physiological benefits, such as anti-inflammatory and stress-relieving properties [13,14]. Emerging evidence also suggests that they may have anti-tumor effects [15]. As VOCs have both harmful and beneficial effects on health, their environmental and biological impacts must be comprehensively investigated [16,17].

As VOCs exert both harmful and beneficial health effects, a clear understanding of their environmental and biological relevance is essential. Indoor TVOC concentrations exhibit considerable variability, with values ranging from 13.5 to 2393.5 μg/m^3^ in Slovak households, where 69% of residences exceeded the recommended threshold of 200 μg/m^3^ [18]. In biological matrices, VOCs are detectable in breath, blood, urine, and buccal swabs, with average detection limits ranging from 3.19 to 13.17 ng using optimized SPME-GC/MS techniques [19]. Moreover, VOCs are actively emitted from the human body itself, with recent human chamber studies reporting emission rates of 286–1030 μg/h from the breath and 164–518 μg/h from whole-body skin, reflecting distinct compound compositions and emission mechanisms—endogenous metabolism predominates in breath VOCs, whereas skin emissions are primarily influenced by exogenous sources [20].

Despite extensive research on VOC emissions, major gaps persist regarding the long-term health effects and combined impacts of multiple VOCs emitted from natural wood [21,22]. Previous studies have predominantly focused on the effects of short-term exposure to individual VOCs, resulting in a significant knowledge gap regarding the cumulative impacts of long-term exposure to complex mixtures of VOCs [23,24,25,26,27]. For example, a study comparing the health effects of Scots pine (*Pinus sylvestris*) and Norway spruce (*Picea abies*) VOCs found no significant differences in outcomes; however, it was limited to short-term exposure and did not account for mixed effects [28]. Moreover, comparative studies on different wood species, particularly those evaluating their potential health benefits, remain unexplored.

This study aimed to fill the current knowledge gaps by thoroughly investigating the VOC emissions from two widely used natural wood species, Korean Red Pine (*Pinus densiflora*) and Japanese Cypress (*Chamaecyparis obtusa*), and their potential biological impacts under controlled experimental conditions. These two species were selected due to their ecological and industrial prominence in South Korean forestry, as well as their documented release of aromatic volatile compounds that may exert bioactive effects relevant to human health. Using a well-established in vivo model, female C57BL/6 mice were exposed to VOCs released from Korean Red Pine and Japanese Cypress for both short (30–60 d) and long (120–180 d) durations. The biological effects of these exposures were extensively evaluated using multi-parameter analysis, including oxidative stress markers, activities of key antioxidant enzymes, hepatic and renal function indicators, and profiles of inflammatory cytokines. By elucidating the dual roles of natural wood VOCs in health and environmental quality, we aimed to provide scientific evidence to support optimized timber usage, enhance indoor air quality management, and inform public health policies.

## 2. Materials and Methods

### 2.1. Investigation of VOC Emissions from Korean Red Pine (Pinus densiflora) and Japanese Cypress (Chamaecyparis obtusa) via HS-GC/MS

The emission of VOCs from Korean Red Pine (*Pinus densiflora*) and Japanese Cypress (*Chamaecyparis obtusa*) was analyzed using headspace gas chromatography–mass spectrometry (HS-GC/MS). To investigate temporal variations in VOC release, wood specimens were collected monthly and analyzed at the Korea Polymer Testing and Research Institute (Seoul, Republic of Korea) using headspace solid-phase microextraction coupled with GC-MS (HS-SPME-GC-MS) under the optimized analytical conditions detailed in Table 1.

### 2.2. Animals and Groupings

**Figure 1:**
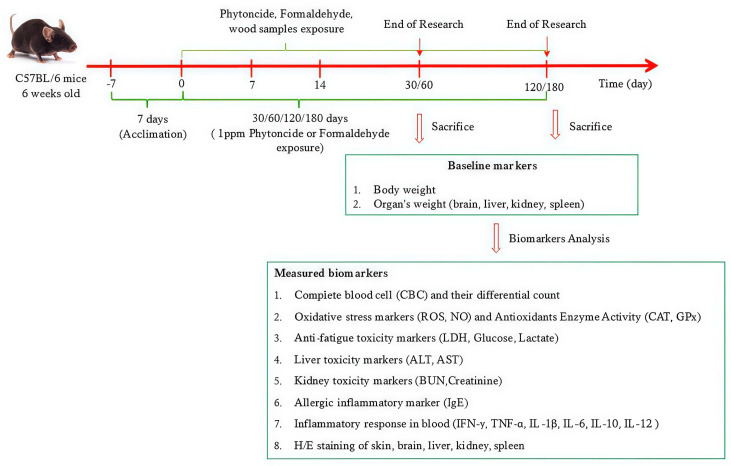
Detailed schematic schedule of experiment.

In total, 120 six-week-old female C57BL/6 mice were purchased from Orient Bio Inc. (Swingman, Seongnam, Republic of Korea), with a mean body weight of 20 ± 1.0 g. The mice were acclimatized for two weeks in a controlled environment at a temperature of 22 ± 2 °C and humidity of 40–60% under a 12:12 h light–dark cycle. Five experimental groups were formed through random assignment, including a control group and four exposure groups: untreated group (NT), phytoncide exposure group (PC), formaldehyde exposure group (NC), Korean Red Pine exposure group (Pine), and Japanese Cypress exposure group (Cypress). Four different time points of 30, 60, 120, and 180 d were used for the study period. Animal experiments were approved by the Institutional Animal Care and Use Committee (IACUC) of Yonsei University Wonju College of Medicine (YWC-230614-1, approval date: 14 June 2024), Republic of Korea. After treatment, mice were humanely euthanized, and whole blood and major organs were collected for the next analysis step. A detailed schematic of the experiment is presented in Figure 1.

### 2.3. Exposure Protocol for Wood Materials and Control Groups

To simulate realistic indoor exposure conditions involving natural wood products, test panels composed of Korean Red Pine and Japanese Cypress, both sourced from domestic manufacturers, were utilized. Each panel was custom trimmed to correspond with the inner sidewalls of the mouse enclosures, while the top and bottom surfaces were deliberately left unsealed to minimize direct animal contact with the materials.

Stock solutions of formaldehyde and phytoncide (10%) were diluted with sterile distilled water to yield a final concentration of 1 ppm. For indirect exposure modeling, 5 mL of each diluted solution was thoroughly combined with wood shavings that had been autoclaved in advance. The treated shavings were then introduced into the mouse cages and systematically renewed once per week to ensure a stable and consistent exposure environment. The same sterilized wood shavings moistened with 5 mL of distilled water were used in the control group under identical housing conditions.

### 2.4. Body Weight Measurements

To obtain baseline data, the body weights of all the mice were measured before beginning the experiment. Subsequently, weekly body weights were measured using a calibrated digital balance adhering to a standardized protocol to minimize handling stress and ensure measurement precision. Each measurement, including the corresponding mouse identification number, date, and weight (g), was systematically recorded.

### 2.5. Histological Examination

Collected organs, including central nervous tissue (brain), metabolic organs (liver and kidney), and lymphoid tissue (spleen), were promptly harvested and fixed in 10% neutral buffered formalin for more than 48 h to ensure optimal preservation of tissue morphology. Following fixation, samples underwent a standard histological preparation protocol, including graded ethanol dehydration, xylene clearing, and paraffin embedding. Paraffin blocks were sectioned at a thickness of 5 μm using a rotary microtome, and the sections were subsequently mounted on glass slides. Hematoxylin and eosin (H&E) staining was performed for microscopic evaluation of histological architecture. Slides were deparaffinized in xylene and rehydrated using a graded ethanol series in distilled water. Hematoxylin was used to stain the nuclei, followed by differentiation in acidic alcohol and bluing in an alkaline solution. Thereafter, the cytoplasm and extracellular matrix were examined, after which the sections were dehydrated, cleared, and covered with coverslips. Histopathological analysis was performed using a fluorescence microscopy platform to evaluate tissue architecture and detect pathological alterations.

### 2.6. Quantification of Total WBC and Differential Leukocyte Populations

Whole blood was obtained via retro-orbital venipuncture and collected into EDTA-coated tubes to prevent coagulation. The samples were gently agitated on an automated tube roller for 5 min to ensure homogenous mixing. Total white blood cell counts and differential leukocyte profiles were subsequently determined using a fully automated hematology analyzer (HEMAVET HV950 FS; Drew Scientific, Erba Diagnostics, Dallas, TX, USA), following the manufacturer’s calibration and operational protocols. This included enumeration of major white blood cell subsets, such as granulocytes (e.g., neutrophils and eosinophils), mononuclear phagocytes (monocytes), and lymphoid-derived cells (lymphocytes).

### 2.7. Evaluation of Serum Total ROS, NO, and Antioxidant Enzyme (GPx and CAT) Activity

Intracellular levels of reactive oxygen species (ROS) were determined through a fluorescence-based approach utilizing 2′,7′-dichlorodihydrofluorescein diacetate (DCFH-DA; Abcam, Cambridge, UK), following the procedural recommendations provided by the supplier. Nitric oxide (NO) levels were analyzed using the Griess reaction-based detection method (Promega, Madison, WI, USA), performed according to the assay-specific documentation. The activities of antioxidant enzymes, including catalase (CAT) and glutathione peroxidase (GPx), were measured with dedicated commercial kits obtained from Biomax Co., Ltd. (Guri, Republic of Korea) and BioVision Inc. (Milpitas, CA, USA). All procedures adhered to standardized protocols provided by each manufacturer and were aligned with validated methodologies described in prior publications [29].

### 2.8. Measurement of Fatigue-Related Metabolic Indicators (LDH, Glucose, and Lactate)

To assess lactate dehydrogenase (LDH) activity in serum, a colorimetric assay kit obtained from BioVision (Milpitas, CA, USA) was employed, and the analysis was carried out according to the supplier’s recommended protocol. To evaluate blood glucose and lactate levels in the serum samples, an assay kit from Biomax Co., Ltd. (Guri, Republic of Korea) was used, following the protocols recommended by the manufacturer. Our methods followed the manufacturer’s instructions and were consistent with a previously published study [30].

### 2.9. Measurement of Serum Hepatic Functions (ALT and AST)

Serum Hepatic Functions (ALT and AST) were quantified using the colorimetric assay kit (Biomax Co., Ltd., Guri, Republic of Korea) in accordance with the protocol provided by the manufacturer. Our methods followed the manufacturer’s instructions and were consistent with a previously published study [30].

### 2.10. Measurement of Serum Renal Functions (BUN, Creatinine)

Quantitative evaluation of blood urea nitrogen (BUN) levels was performed using an assay system provided by Abbexa Ltd. (Cambridge, UK), following the standard procedures outlined in the kit’s documentation. Serum creatinine analysis was carried out with a separate commercial kit from the same supplier, adhering closely to the established operational protocol. Both assays were conducted in line with the supplier’s instructions and aligned with the methodology previously reported in the literature [30].

### 2.11. Measurement of Serum IgE Levels

Serum IgE levels were determined using a mouse IgE ELISA Kit (Abcam, Cambridge, UK) as per the manufacturer’s instructions. The IgE level was determined by measuring absorbance at 450 nm using a SpectraMax^®^ ABS Plus microplate reader (Molecular Devices, San Jose, CA, USA).

### 2.12. Serum Cytokines Analysis

Serum concentrations of representative pro- and anti-inflammatory cytokines, such as IFN-γ, TNF-α, IL-1β, IL-6, IL-10, and IL-12 (p70), were quantified using a multiplexed bead-based immunoassay platform (Bio-Rad, San Diego, CA, USA), following the experimental procedures provided by the supplier. Fluorescence signal acquisition and data processing were conducted using the Bio-Plex 200 analytical system (Luminex, Austin, TX, USA). Cytokine concentrations were derived from standard curves generated via a five-parameter logistic (5-PL) regression model to ensure accurate and reliable quantification.

### 2.13. Statistical Analysis

All data are presented as mean ± SEM. One-way ANOVA followed by Tukey’s post hoc test was performed using GraphPad Prism (version 10.0; GraphPad Software Inc., La Jolla, CA, USA). Adjusted *p*-values accounting for multiple comparisons were automatically calculated by the software. A threshold of *p* < 0.05 was considered statistically significant.

## 3. Results

### 3.1. GC–MS-Based VOCs Profiling of Korean Red Pine (Pinus densiflora) and Japanese Cypress (Chamaecyparis obtusa)

Based on the analytical data obtained from the Korea Polymer Testing and Research Institute, we performed a time-resolved profiling of VOC emissions from each wood type at four designated intervals. To ensure chemical nomenclature accuracy and consistency, all detected VOCs were validated against the PubChem database: “(https://pubchem.ncbi.nlm.nih.gov, accessed on 5 April 2025)”. For each compound, core physicochemical and toxicological attributes were annotated, such as human toxicity, flammability, and explosion risk. Additionally, their potential biological effects were explored through a targeted literature search using PubChem resources. Compounds were subsequently categorized into positive and negative VOCs based on their reported health and environmental impacts. Their emission levels were semi-quantitatively expressed as relative area percentages for comparative analysis. Complete datasets are provided in Supplementary Tables S1–S5.

GC–MS profiling demonstrated that Japanese Cypress (*Chamaecyparis obtusa*) emitted a greater number of VOCs across all assessed time points (65, 71, 71, and 80 compounds at 30, 60, 120, and 180 days, respectively) in comparison to Korean Red Pine (*Pinus densiflora*), which released 36, 46, 50, and 58 compounds over the same intervals. These results suggest that Japanese Cypress possesses a more chemically diverse emission profile throughout the aging process.

Based on annotations from the PubChem database, all identified VOCs were categorized into three groups: positive VOCs (compounds with reported beneficial or non-toxicological profiles), negative VOCs (compounds associated with documented toxicological or environmental risks), and unclassified VOCs for which no toxicological data or reference information was available. Over the 30-, 60-, 120-, and 180-day assessment period, the relative abundance of positive VOCs in Korean Red Pine decreased progressively from 82.40% to 82.20%, 79.73%, and 73.40%, respectively. Similarly, Japanese Cypress exhibited a comparable declining trend, with values of 74.10%, 73.60%, 74.04%, and 69.00% at the same time points.

In contrast, the proportion of negative VOCs followed a biphasic pattern in both species. In Korean Red Pine, negative VOCs increased from 5.30% at 30 days to 8.70% at 60 days and then slightly decreased to 6.32% at 120 days, followed by a marked increase to 13.10% at 180 days. Japanese Cypress showed a similar but less pronounced fluctuation, with corresponding values of 2.00%, 2.20%, 1.80%, and 4.00%, respectively.

Notably, while the profiles of positive VOCs were relatively comparable between the two wood types, Japanese Cypress consistently exhibited lower proportions of negative VOCs throughout the study period. This difference was especially evident at 180 days, where the negative VOC proportion in Korean Red Pine (13.10%) was more than threefold higher than that in Japanese Cypress (4.00%).

Further compositional analysis revealed clear differences in the dominant VOC species released by the two wood types. In Korean Red Pine, the most abundant positive VOCs included alpha-pinene, longifolene, alpha-terpineol, benzaldehyde, acetic acid, and D-limonene. The major negative VOCs detected were methanol, hexanal, (+)-sativene, o-isopropenyltoluene, and acetone. In contrast, Japanese Cypress emitted a different set of dominant VOCs, with alpha-pinene, alpha-cadinol, alpha-muurolene, tau-cadinol, alpha-terpineol, and D-limonene constituting the top positive compounds. The predominant negative VOCs identified in Japanese Cypress were methanol, acetone, furfural, and o-isopropenyltoluene, reflecting a less diverse and potentially less harmful toxicological profile compared to Korean Red Pine.

These findings indicate that, despite exhibiting comparable profiles of favorable VOC emissions, Japanese Cypress shows greater potential to exert a lower toxicological impact and confers beneficial biological effects during prolonged exposure.

### 3.2. Effects of Natural Wood Sample Exposure on the Weights of Body and Major Organs in Female C57BL/6 Mice

**Figure 2:**
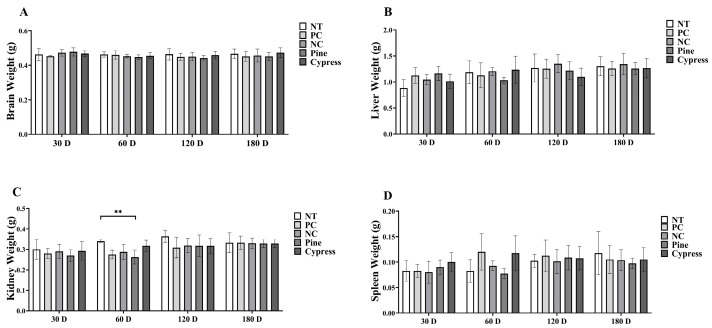
Effects of exposure to Korean Red Pine and Japanese Cypress on major organ weight in female C57BL/6 mice. (**A**) Brain; (**B**) liver; (**C**) kidney; (**D**) spleen. Data are presented as mean ± SEM. ** *p* < 0.01 indicates statistical difference when measured with one-way ANOVA. Abbreviations: NT, non-treated control; PC: phytoncide exposure group; NC: formaldehyde exposure group; Pine: Korean Red Pine wood exposure group; Cypress: Japanese Cypress wood exposure group. These abbreviations apply to all the following figures and tables.

To evaluate the physiological impact of wood-derived VOC exposure, body mass and internal organ weights, including the brain, liver, spleen, and kidneys, were measured in female C57BL/6 mice at 30, 60, 120, and 180 days. Throughout the experimental period, body weights remained consistent across all exposure groups, with no statistically meaningful variations detected between the baseline and the final time point (Supplementary Figure S1). Similarly, comparative analyses of organ weights showed no notable differences in the brain, liver, or spleen among the various groups. However, kidney weights were significantly higher in the non-treated (NT) group than in the pine-treated group at 60 d (Figure 2). This isolated difference suggests that wood samples had minimal physiological impact, with limited organ-specific variations. Overall, long-term exposure to wood samples did not significantly affect body or organ weights, supporting the general safety of the tested materials under the study conditions.

### 3.3. Effects of Natural Wood Sample Exposure on Histopathological Analysis of Major Organs in Female C57BL/6 Mice

Histopathological assessments of major organs were conducted using hematoxylin and eosin (H&E) staining to evaluate potential tissue-level toxicity following exposure to wood products. No significant morphological alterations, inflammatory cell infiltration, or tissue damage were observed across all experimental groups, including the NT, phytoncide-treated (PC), and formaldehyde-treated negative control (NC) groups. Such changes were also not observed from 30 to 180 days of exposure to wood samples (Supplementary Figures S2–S6). These results indicate that prolonged exposure to Korean Red Pine and Japanese Cypress-derived compounds causes no detectable histopathological changes, supporting the conclusion that the tested wood products had minimal adverse effects on organ integrity under the study conditions.

### 3.4. Effects of Natural Wood Sample Exposure on Complete Blood Cell Count in Female C57BL/6 Mice

**Figure 3:**
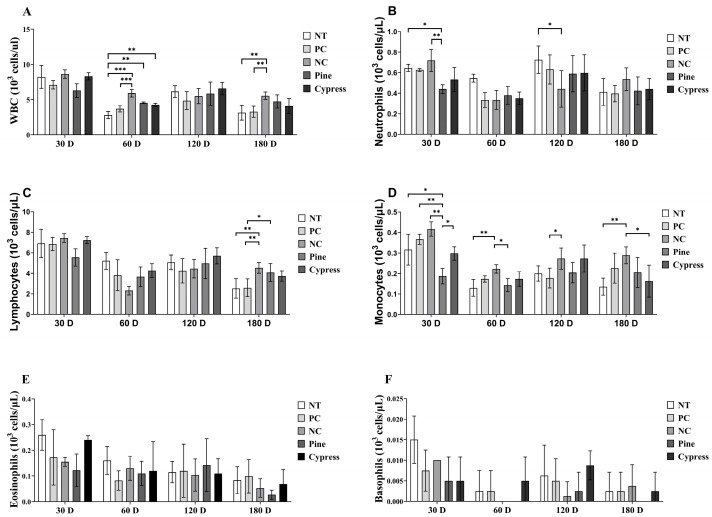
Effects of exposure to Korean Red Pine and Japanese Cypress on total WBC counts and differential leukocyte populations in female C57BL/6 mice. (**A**) Total WBC count; (**B**) neutrophil count; (**C**) lymphocyte count; (**D**) monocyte count; (**E**) eosinophil count; (**F**) basophil count. Data are presented as mean ± SEM from three independent experiments. Statistical significance was determined using one-way ANOVA. * *p* < 0.05, ** *p* < 0.01, and *** *p* < 0.001 indicate statistical differences when measured with one-way ANOVA.

White blood cells (WBCs) are essential markers of immune function and immunotoxicity in response to environmental exposures [31]. In this study, WBC profiles, including total leukocytes and their subtypes, were evaluated in female C57BL/6 mice following short-term (30–60 d) and long-term (120–180 d) exposure to wood samples. Compared with the Korean Red Pine and Japanese Cypress groups, the NC group exhibited significantly elevated levels of total WBCs, neutrophils, and monocytes at multiple time points (Figure 3). Conversely, no significant differences were observed in lymphocyte, eosinophil, or basophil counts among the treatment groups. Notably, neutrophil and monocyte counts demonstrated temporal fluctuations, with a decrease at 60 d, a peak at 120 d, and a subsequent decline at 180 d, compared to the 30 d values. These findings suggest that exposure to wood-derived compounds selectively modulates specific components of the innate immune system, particularly neutrophils and monocytes, without affecting all leukocyte subtypes. The observed temporal variations highlight the dynamic nature of the immune responses to wood exposure and warrant further investigation into its long-term immunological effects.

### 3.5. Effects of Wood Exposure on Redox Balance and Antioxidant Defenses in C57BL/6 Female Mice

**Figure 4:**
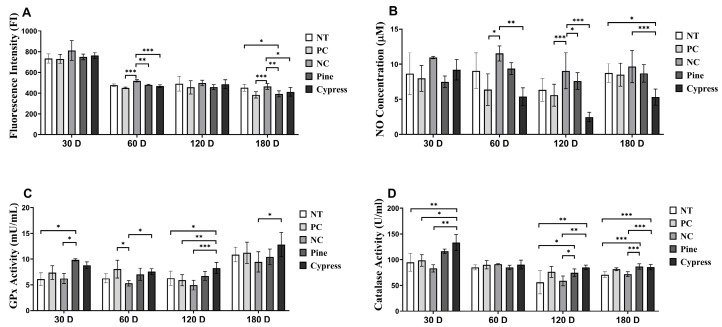
Effects of Korean Red Pine and Japanese Cypress exposure on redox balance and antioxidant defense markers of female C57BL/6 mice. (**A**) Total serum ROS level; (**B**) serum NO level; (**C**) serum GPx level; (**D**) serum catalase level. Data are presented as mean ± SEM from three independent experiments. * *p* < 0.05, ** *p* < 0.01, and *** *p* < 0.001 indicate statistical difference when measured with one-way ANOVA.

Reactive oxygen species (ROS) and nitric oxide (NO) are widely recognized as biomarkers of systemic toxicity and are often elevated in response to environmental stress. Excessive ROS generation can disrupt the cellular redox homeostasis and lead to metabolic disturbances, inflammation, and disease development [32]. In the present study, the ROS levels remained unchanged across all treatment groups after 30 d. However, after 60 d, the NC group exhibited significantly elevated ROS levels compared with the Korean Red Pine and Japanese Cypress groups. Notably, at 180 d, the ROS levels in the Korean Red Pine and Japanese Cypress groups were significantly lower than those in the untreated and formaldehyde exposure groups, indicating a potential alleviation of redox imbalance associated with prolonged wood exposure. No significant differences in NO production were observed between the groups at 30 or 60 d. However, at 120 and 180 d, the Japanese Cypress extract group exhibited significantly reduced NO levels compared with the NC group, indicating a possible downregulation of NO-associated inflammatory signaling with extended exposure. Moreover, glutathione peroxidase (GPx) activity showed no differences among different treatment groups after 60 d. After 30 d, the Korean Red Pine group displayed significantly higher GPx levels than those in the NC group, whereas at both 120 and 180 d, the Japanese Cypress group exhibited significantly elevated GPx activity relative to that in the NC group. Catalase activity remained stable across all groups at 60 and 180 d; however, it was significantly higher in the Korean Red Pine (*Pinus densiflora*) group at 30 d and in both the Korean Red Pine (*Pinus densiflora*) and Japanese Cypress groups at 120 d than that in the NC group. Collectively, these findings indicate that long-term exposure to certain wood products, particularly Korean Red Pine (*Pinus densiflora*) and Japanese Cypress, may attenuate oxidative stress by enhancing endogenous antioxidant defense mechanisms. These results provide mechanistic insights into the redox-modulatory effects of wood products (Figure 4).

### 3.6. Effects of Natural Wood Sample Exposure on Toxicity Markers in Female C57BL/6 Mice

**Figure 5:**
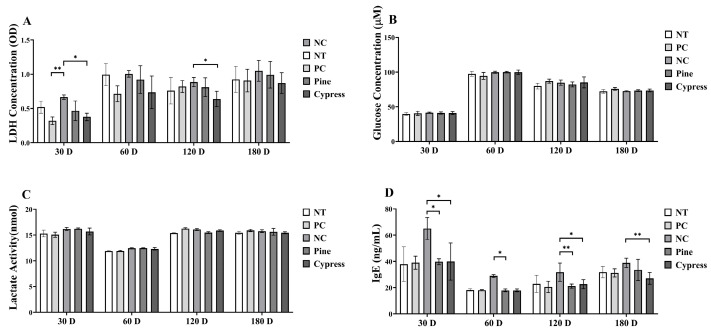
Impact of exposure to Korean Red Pine and Japanese Cypress on the toxicity markers of female C57BL/6 mice. (**A**): LDH activity; (**B**): serum glucose level; (**C**): serum lactate level; (**D**): Serum IgE level. Data are presented as mean ± SEM from three independent experiments. * *p* < 0.05 and ** *p* < 0.01 indicate statistical differences when measured with one-way ANOVA.

In this study, toxicity-related biomarkers were evaluated in female C57BL/6 mice exposed to Korean Red Pine and Japanese Cypress wood samples over periods ranging from 30 to 180 d, with the aim of assessing the physiological impact of naturally emitted compounds from wood products. The key endpoints included markers of fatigue, hepatic and renal function, and immune response (Figure 5). First, lactate levels, an indicator of fatigue, were significantly reduced in the Japanese Cypress extract group at 120 d compared to the NC group, whereas no significant differences were observed at other time points (Figure 5C). Lactate dehydrogenase (LDH) activity was significantly lower in the Japanese Cypress group at 30 and 60 d; however, it did not differ among groups at 120 and 180 d (Figure 5A). The glucose levels remained consistent across all groups throughout the study period (Figure 5B). Moreover, immune function analysis revealed that immunoglobulin E (IgE) levels significantly decreased in the Korean Red Pine and Japanese Cypress exposure groups at 30 and 120 d, with the Japanese Cypress group maintaining significantly lower IgE levels after 180 d (Figure 5D).

**Figure 6:**
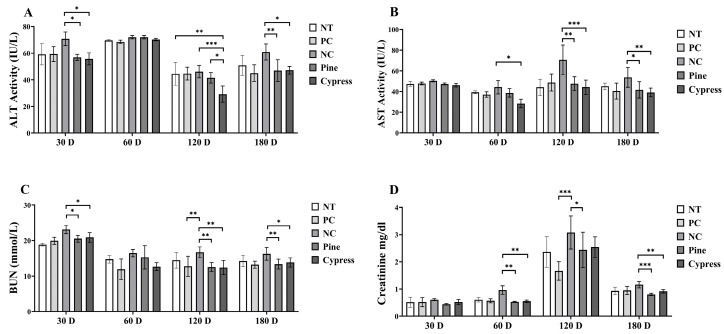
Effect of treatment of Korean Red Pine and Japanese Cypress on the toxicity markers of female C57BL/6 mice. (**A**): Serum ALT; (**B**): serum AST; (**C**): serum BUN; (**D**): serum creatinine. Data are presented as mean ± SEM from three independent experiments. * *p* < 0.05, ** *p* < 0.01, and *** *p* < 0.001 indicate statistical differences when measured with one-way ANOVA.

Hepatic function was assessed by measuring serum alanine aminotransferase (ALT) and aspartate aminotransferase (AST) levels. ALT levels were significantly reduced in both the Korean Red Pine and Japanese Cypress treatment groups at 30 and 180 d, with the Japanese Cypress treatment group showing the lowest ALT values at 120 d (Figure 6A). AST levels were significantly lower in the Japanese Cypress group at 60 d and in both the Korean Red Pine and Japanese Cypress groups at 120 and 180 d compared to the NC group (Figure 6B). Additionally, renal function, evaluated using blood urea nitrogen (BUN) and creatinine levels, was consistently lower in the Korean Red Pine and Japanese Cypress groups at various time points. Notably, the creatinine levels were significantly reduced in the pine treatment group after 120 d (Figure 6C,D). Collectively, these findings suggest that exposure to VOCs from Korean Red Pine and Japanese Cypress wood products may exert protective effects by reducing the biochemical markers of systemic toxicity, improving liver and kidney function, modulating immune responses, and potentially alleviating fatigue.

### 3.7. Effects of Natural Wood Sample Exposure on Cytokine Level in Female C57BL/6 Mice

**Figure 7:**
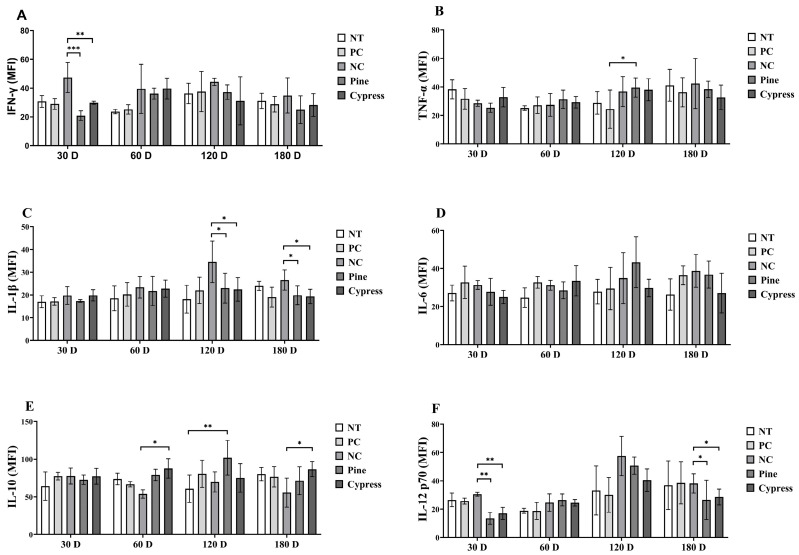
Effects of exposure to Korean Red Pine and Japanese Cypress on cytokines in female C57BL/6 mice. (**A**): Serum IFN-γ level; (**B**): serum TNF- α level; (**C**): serum IL-1β level; (**D**): serum IL-6 v; (**E**): serum IL-10 level; (**F**): serum IL-12 (p70) level. Data are presented as mean ± SEM from three independent experiments. * *p* < 0.05, ** *p* < 0.01, and *** *p* < 0.001 indicate statistical difference when measured with one-way ANOVA.

Cytokines are critical regulators of immune function that mediate intercellular communication and orchestrate inflammatory responses [33]. Key cytokines include interferon-gamma (IFN-γ), which activates macrophages and promotes Th1-mediated antiviral and anti-tumor responses [34]; interleukin-1 beta (IL-1β), which enhances immune cell recruitment and vascular permeability [35]; interleukin-6 (IL-6), which mediates B cell differentiation and acute-phase responses [36]; tumor necrosis factor-alpha (TNF-α), a central pro-inflammatory cytokine involved in immune activation and apoptosis; and interleukin-10 (IL-10), which exerts anti-inflammatory effects by suppressing Th1 cytokine production [37,38]. In the present study, IFN-γ levels were significantly reduced in the Korean Red Pine and Japanese Cypress treatment groups compared to the NC group at 30 d; however, no significant differences were observed across the treatment groups at 60, 120, or 180 d (Figure 7A). TNF-α levels remained stable across most time points, except at 60 d, when the Korean Red Pine group exhibited significantly higher levels than those in the PC group (Figure 7B). IL-1β concentrations were significantly elevated in the NC group compared to the Korean Red Pine and Japanese Cypress groups at 120 and 180 d, suggesting that formaldehyde exposure is associated with pro-inflammatory effects (Figure 7C). IL-6 levels remained consistent across all groups throughout the study period, indicating that it likely plays a limited role in the observed immune modulation (Figure 7D). IL-10, an anti-inflammatory cytokine, was significantly increased in the Japanese Cypress group at 60 d and in the pine group at 120 d compared to the NC group, suggesting the potential immunoregulatory effects of wood product exposure (Figure 7E). IL-12 (p70) levels were significantly reduced in the Korean Red Pine and Japanese Cypress groups at 30 and 180 d compared with those in the NC group, whereas no differences were observed at 60 and 120 d (Figure 7F). Overall, these findings suggest that exposure to VOCs from Korean Red Pine and Japanese Cypress wood products modulates specific pro- and anti-inflammatory cytokine profiles, with potential implications for immune homeostasis and inflammation regulation during prolonged exposure.

## 4. Discussion

Korean Red Pine (*Pinus densiflora*) is a major temperate coniferous tree species primarily distributed in Northeast Asia, including northeastern China, the Korean Peninsula, the Russian Far East, and northern Japan. In South Korea, the Korean Red Pine is the predominant forest species, accounting for approximately 37% of the forested areas of the country [39]. Similarly, Japanese Cypress (*Chamaecyparis obtusa*), a widely distributed genus of coniferous trees, is known for its aromatic and durable wood. Commonly found in temperate and subtropical regions, Japanese Cypress has historically been valued for its resistance to decay and its potential applications in traditional medicine and environmental settings [40,41].

In this study, we assessed the in vivo toxicological effects of VOCs from Korean Red Pine and Japanese Cypress on a spectrum of health-related biomarkers in C57BL/6 mice. Toxicity was evaluated based on inflammation, acute-phase response, oxidative stress levels, and histopathological changes. Previous studies have highlighted that exposure to VOCs in the environment via ingestion and skin contact is a key health concern because of their adverse effects on human health. Furthermore, such exposure has been associated with a wide range of diseases, from mild irritation to severe conditions such as cancer [42].

Internal organs, such as the liver, kidney, spleen, and brain, play critical roles in forensic medicine and pathology, as changes in their weight and size can signal abnormalities. Oxidative stress and chronic inflammation can disrupt the normal function and structure of organs, resulting in significant changes [43,44]. In our study, no visible pathological anomalies or abnormalities in the primary organs were observed after wood treatment when compared to the control groups. This indicated that wood treatment did not induce major detrimental effects or structural changes in these essential organ systems.

The transient reduction in total leukocyte and differential counts observed at the 60-day exposure point may reflect a phase of immune modulation induced by cumulative VOC exposure. Both wood types emitted high levels of bioactive monoterpenes, including α-pinene and D-limonene, which have been previously reported to inhibit T-cell proliferation, reduce cytokine production, and modulate immune activation pathways [15,45]. Such effects may account for the observed leukocyte suppression, potentially via oxidative stress-related signaling or feedback regulation. The subsequent normalization of cell counts at later time points (120–180 days) suggests the onset of physiological adaptation.

Inflammation influences both ROS and NO levels and may contribute to VOC generation. Inflammatory processes can enhance oxidative stress, which, in turn, affects VOC production [13,46,47,48]. Wood treatment in mice did not significantly influence oxidative stress levels compared with the NT group. Moreover, the wood-treated group exhibited significantly reduced levels of oxidative stress markers compared to the NC group, suggesting that the treatment was safe. Additionally, the wood-treated group showed enhanced levels of antioxidants, such as GPx and catalase, which counteracted oxidative stress.

Pre-clinical investigations often encounter challenges because the commonly used blood indicators may fail to detect subtle changes in renal function. Typically, serum creatinine levels do not increase until approximately 50% of the renal function is lost [49,50]. We also examined renal function in relation to VOC exposure. The results showed that the BUN and creatinine levels in the wood-treated group remained consistently within normal physiological ranges, in contrast to those in the control groups. These findings highlighted the preservation of renal health and function in the wood-treated group.

LDH is a blood marker of cellular and tissue damage. Mild hypoglycemia can result from gluconeogenic organ dysfunction, which has been linked to impaired renal and liver function; this can significantly affect prognosis [51]. In our study, wood treatment in mice did not cause a statistically significant increase in serum biochemical markers compared to the control groups. Although some LDH levels are normal, elevated levels have been associated with various disorders and diseases [52]. We specifically examined the LDH levels after VOC exposure and their potential health consequences. The LDH levels in the wood treatment group were closely aligned with those observed in the NT group. This finding emphasizes the absence of statistically significant changes in LDH activity owing to wood treatment, indicating a state of LDH homeostasis that is comparable to that of the untreated control group. Moreover, high levels of glucose and lactate can overwhelm the antioxidant defenses of the body, thereby contributing to oxidative stress [53]. Such oxidative stress may alter VOC synthesis and release, which has implications for monitoring and understanding the oxidative stress of an individual in relation to various health conditions.

Liver biomarkers, such as ALT and AST, are critical indicators of liver dysfunction, with elevated levels often signifying liver damage or inflammation [54,55]. VOC exposure has been associated with various biological responses, including hepatic injury, which is characterized by rapid alterations in liver markers. Elevated levels of these markers indicate potential liver damage. In this study, we assessed the liver function in C57BL/6 mice by examining the effects of a 30 d wood treatment regimen on these markers. The findings revealed a significant reduction in AST activity in the Korean Red Pine and Japanese Cypress groups compared to that in the NC group, indicating an ameliorative effect on liver function. Similarly, ALT activity was notably diminished in the Korean Red Pine and Japanese Cypress groups, further underscoring the beneficial effects of treatment on liver health.

Inflammation and oxidative stress can exacerbate the symptoms of chronic conditions, such as sickle cell disease, and may trigger VOC episodes [56]. In our study, wood treatment effectively reduced oxidative stress and inflammation. Furthermore, atopic disorders, parasitic diseases, cutaneous disorders, neoplastic diseases, and immune deficiencies have been associated with elevated blood IgE levels [57]. Notably, the wood treatment group exhibited a robust immunological response, as evidenced by IgE levels comparable to those of the normal control group. These findings strongly suggest that wood treatment did not cause any substantial adverse effects on the overall health or immunological reactivity of the mice.

Cytokine analyses indicated that Korean Red Pine and Japanese Cypress treatments modulated immune responses by balancing pro- and anti-inflammatory cytokines. The significant reduction in IL-1β and TNF-α at later stages (120 and 180 d) indicates that Korean Red Pine and Japanese Cypress exposure exhibited the potential to modulate inflammatory cytokines. Previous studies have attributed this effect to bioactive compounds such as α-pinene, which suppress inflammation by inhibiting the NF-κB signaling pathway [58,59]. IL-10 is crucial in suppressing excessive immune responses and maintaining immune homeostasis [38]. In our study, IL-10 levels increased after 60 and 120 d, indicating that Korean Red Pine and Japanese Cypress extracts enhanced anti-inflammatory responses. This could likely be attributed to the fact that plant-derived terpenoids are associated with enhanced IL-10 production [60]. IL-12 is a key driver of IFN-γ production due to its role in promoting Th1-type immune responses [61]. Our data showed a decrease in IL-12 and IFN-γ levels at specific time points, indicating that Korean Red Pine and Japanese Cypress treatments can reduce pro-inflammatory activity, likely by regulating Th1-type immune responses. This coordinated regulation of cytokines demonstrates the potential of Korean Red Pine and Japanese Cypress extracts to mitigate inflammation by reducing pro-inflammatory markers and enhancing anti-inflammatory pathways. These findings highlight the immunomodulatory potential of Korean Red Pine and Japanese Cypress wood treatments and their therapeutic value in the management of inflammatory conditions.

Corroborating the measured physiological and immunological changes, GC–MS profiling revealed distinct temporal and compositional differences in the VOCs emitted by the two wood types. Notably, Japanese Cypress consistently emitted a higher number of VOC species than Korean Red Pine across all aging time points, suggesting a richer and potentially more diverse reservoir of bioactive constituents. Despite this greater chemical complexity, the relative abundance of negative VOCs, compounds associated with toxicological or environmental risks, remained consistently lower in Japanese Cypress, particularly at the 180-day mark (4.00% vs. 13.10% in Korean Red Pine). This compositional divergence may account for the more favorable biological outcomes observed in the Japanese Cypress group, including attenuated oxidative stress, enhanced antioxidant enzyme activity, and a more balanced cytokine profile. Among the dominant positive VOCs identified, such as alpha-pinene, D-limonene, and alpha-terpineol, several have been previously demonstrated to modulate immune responses and suppress oxidative damage via NF-κB inhibition and reactive oxygen species (ROS) scavenging, as well as through modulation of Ras–ERK signaling pathways and promotion of antioxidant defenses such as GPx and catalase [58,62]. Conversely, the transient leukocyte reduction observed at 60 days may be partially attributed to short-term immunosuppressive effects of specific VOCs, while the later-phase recovery suggests metabolic adaptation or selective clearance of pro-inflammatory compounds. Overall, these findings highlight that the ratio and persistence of beneficial versus detrimental VOCs, rather than their total emission load, critically shape the systemic biological responses to long-term wood exposure.

While GC–MS profiling provided detailed insights into the VOC composition of the raw wood materials, we acknowledge that the actual VOC exposure environment within the mouse cages may have involved contributions from non-wood sources, such as feed, urine, and feces. Due to the anticipated and unavoidable background interference from these sources, in-cage GC-MS sampling was not conducted, representing a methodological limitation of the present study. This constraint limited our ability to definitively attribute the observed physiological and immunological responses solely to wood-derived VOCs. Nonetheless, the concordance between the known bioactivity of major VOC constituents and the observed reductions in oxidative stress, inflammatory cytokines, and immune modulation provides mechanistic plausibility for a causal relationship. Future investigations utilizing refined in-cage sampling strategies, VOC-isolated exposure chambers, or compound-specific tracing approaches will be essential for delineating the precise contributions of individual VOCs to the observed systemic effects.

## 5. Conclusions

This study evaluated the effects of VOCs emitted by Korean Red Pine (*Pinus densiflora*) and Japanese Cypress (*Chamaecyparis obtusa*) wood-emitted VOCs on key physiological and biochemical markers in C57BL/6 mice. The results revealed significant reductions in pro-inflammatory cytokines (IL-1β, TNF-α, IL-12, and IFN-γ) and oxidative stress markers, along with increased levels of the anti-inflammatory cytokine IL-10 and antioxidant enzymes, such as GPx and catalase. However, no pathological abnormalities were observed in the major organs, liver function markers (ALT and AST), and renal function markers (BUN and creatinine), highlighting the safety of these treatments. Complementary GC–MS analysis revealed that Japanese Cypress released a greater number of VOCs at all measured time points and consistently exhibited a lower proportion of negative VOCs compared to Korean Red Pine, particularly at 180 days (4.00% vs. 13.10%), indicating a more favorable chemical emission profile. These findings highlight the potential of Korean Red Pine and Japanese Cypress wood treatments in regulating inflammation, mitigating oxidative stress, and preserving overall physiological health. Our findings establish the therapeutic value and suitability of these treatments for avoiding environments with adverse health impacts, thereby allowing sustainable applications of VOCs.

## Figures and Tables

**Table 1:** Instrument parameters for HS-GC/MS analysis of VOC emissions.

Parameter	Setting/Specification
Headspace Sampler	Agilent 7697A (Agilent Technologies, Santa Clara, CA, USA)
Incubation temperature	150 °C
Incubation time	30 min
GC-MS system	Agilent 8890 GC coupled with Agilent 5977C MSD (Agilent Technologies, Santa Clara, CA, USA)
GC column	Agilent J&W DB-624 (30 m × 0.25 mm × 1.4 µm)
Carrier gas	Helium (≥99.999% purity)
Carrier gas flow	1.5 mL/min (constant flow)
Ionization mode	Electron ionization (EI)
Ionization energy	70 eV
Mass scan range	29–550 m/z

## Data Availability

All data analyzed in this study are included in this article.

## Supplementary Materials

The following supporting information can be downloaded at https://www.mdpi.com/article/10.3390/toxics13080650/s1. Figure S1: Short-term 30–60 days and Long-term 120–180 days effects on body weight of VOCs released from wood samples in the mice cages; Figure S2: The H&E Staining of Skin; Figure S3: The H&E Staining of Brian. Figure S4: The H&E Staining of Liver. Figure S5: The H&E Staining of Kidney. Figure S6: The H&E Staining of Spleen. Table S1: Total Number of VOCs and Area Percentage Change of Positive and Negative VOC Release Areas from Korea Red Pine (*Pinus densiflora*) and Japanese Cypress (*Chamaecyparis obtusa*) over 30–180 Days; Table S2: Main Positive VOCs and Trend Analysis for Korea Red Pine (*Pinus densiflora*) over 30–180 Days; Table S3: Main Negative VOCs and Trend Analysis for Korea Red Pine (*Pinus densiflora*) over 30–180 Days; Table S4: Main Positive VOCs and Trend Analysis for Japanese Cypress (*Chamaecyparis obtusa*) over 30–180 Days; Table S5: Main Negative VOCs and Trend Analysis for Japanese Cypress (*Chamaecyparis obtusa*) over 30–180 Days.

## Abbreviations

The following abbreviations are used in this manuscript:

ALT	Alanine aminotransferase;
AST	Aspartate aminotransferase;
BUN	Blood urea nitrogen;
DCFH-DA	Dichlorodihydrofluorescein diacetate;
GPx	Glutathione peroxidase;
H&E	Hematoxylin and eosin;
IFN-γ	Interferon gamma;
IgE	Immunoglobulin E;
IL-1β	Interleukin 1 beta;
IL-10	Interleukin 10;
IL-6	Interleukin 6;
NC	Negative control (formaldehyde-treated);
NO	Nitric oxide;
NT	Non-treated;
OD	Optical density;
OS	Oxidative stress;
PC	Phytoncide-treated;
ROS	Reactive oxygen species;
TNF-α	Tumor necrosis factor alpha;
VOCs	Volatile organic compounds;
WBCs	White blood cells;
GC-MS	Gas chromatography–mass spectrometry.

## References

[1] Anand S., Philip B., Mehendale H. (2014). Volatile organic compounds. Encycl. Toxicol..

[2] Koppmann R. (2020). Chemistry of volatile organic compounds in the atmosphere. Hydrocarbons, Oils and Lipids: Diversity, Origin, Chemistry and Fate.

[3] Hester R.E., Harrison R.M. (1995). Volatile Organic Compounds in the Atmosphere.

[4] WHO (1989). Indoor Air Quality: Organic Pollutants: Report on a WHO Meeting, Berlin (West), 23–27 August 1987.

[5] Panagopoulos I.K., Karayannis A.N., Kassomenos P., Aravossis K. (2011). A CFD simulation study of VOC and formaldehyde indoor air pollution dispersion in an apartment as part of an indoor pollution management plan. Aerosol Air Qual. Res..

[6] Burroughs H., Hansen S.J. (2020). Managing Indoor Air Quality.

[7] Sterling D.A. (2018). Volatile organic compounds in indoor air: An overview of sources, concentrations, and health effects. Indoor Air and Human Health.

[8] Roffael E. (2006). Volatile organic compounds and formaldehyde in nature, wood and wood based panels. Holz Als Roh-Und Werkst..

[9] Adamová T., Hradecký J., Pánek M. (2020). Volatile organic compounds (VOCs) from wood and wood-based panels: Methods for evaluation, potential health risks, and mitigation. Polymers.

[10] Jones A.P. (1999). Indoor air quality and health. Atmos. Environ..

[11] Khoshakhlagh A.H., Mohammadzadeh M., Manafi S.S., Yousefian F., Gruszecka-Kosowska A. (2023). Inhalational exposure to formaldehyde, carcinogenic, and non-carcinogenic risk assessment: A systematic review. Environ. Pollut..

[12] IARC Working Group on the Evaluation of Carcinogenic Risks to Humans (2006). Formaldehyde, 2-butoxyethanol and 1-tert-butoxypropan-2-ol. IARC Monogr. Eval. Carcinog. Risks Hum..

[13] Salehi B., Upadhyay S., Erdogan Orhan I., Kumar Jugran A., Jayaweera S.L.D., Dias A.D., Sharopov F., Taheri Y., Martins N., Baghalpour N. (2019). Therapeutic potential of α- and β-pinene: A miracle gift of nature. Biomolecules.

[14] Shabani M., Erfani S., Abdolmaleki A., Afzali F.E., Khoshnazar S.M. (2023). Alpha-pinene modulates inflammatory response and protects against brain ischemia via inducible nitric oxide synthase-nuclear factor-kappa B-cyclooxygenase-2 pathway. Mol. Biol. Rep..

[15] Abe M., Asada N., Kimura M., Fukui C., Yamada D., Wang Z., Miyake M., Takarada T., Ono M., Aoe M. (2024). Antitumor activity of α-pinene in T-cell tumors. Cancer Sci..

[16] Zhou X., Zhou X., Wang C., Zhou H. (2023). Environmental and human health impacts of volatile organic compounds: A perspective review. Chemosphere.

[17] Antonelli M., Donelli D., Barbieri G., Valussi M., Maggini V., Firenzuoli F. (2020). Forest volatile organic compounds and their effects on human health: A state-of-the-art review. Int. J. Environ. Res. Public Health.

[18] Mečiarová Ľ., Vilčeková S., Krídlová Burdová E., Kiselák J. (2017). Factors effecting the total volatile organic compound (TVOC) concentrations in Slovak households. Int. J. Environ. Res. Public Health.

[19] Kusano M., Mendez E., Furton K.G. (2011). Development of headspace SPME method for analysis of volatile organic compounds present in human biological specimens. Anal. Bioanal. Chem..

[20] Zou Z., Yang X. (2022). Volatile organic compound emissions from the human body: Decoupling and comparison between whole-body skin and breath emissions. Build. Environ..

[21] Kovačević M., Rieder-Gradinger C., Teischinger A., Srebotnik E. (2023). Volatile organic compounds emitted from Scots pine and Norway spruce wood. Eur. J. Wood Prod..

[22] Junge K.M., Buchenauer L., Elter E., Butter K., Kohajda T., Herberth G., Röder S., Borte M., Kiess W., von Bergen M. (2021). Wood emissions and asthma development: Results from an experimental mouse model and a prospective cohort study. Environ. Int..

[23] Leal M.P., Brochetti R.A., Ignácio A., Câmara N.O.S., da Palma R.K., de Oliveira L.V.F., de Fátima Teixeira da Silva D., Lino-Dos-Santos-Franco A. (2018). Effects of formaldehyde exposure on the development of pulmonary fibrosis induced by bleomycin in mice. Toxicol. Rep..

[24] Lang I., Bruckner T., Triebig G. (2008). Formaldehyde and chemosensory irritation in humans: A controlled human exposure study. Regul. Toxicol. Pharmacol..

[25] Wei C., Chen M., You H., Qiu F., Wen H., Yuan J., Xiang S., Yang X. (2017). Formaldehyde and co-exposure with benzene induce compensation of bone marrow and hematopoietic stem/progenitor cells in BALB/c mice during post-exposure period. Toxicol. Appl. Pharmacol..

[26] Waidyanatha S., Hackett M., Black S.R., Stout M.D., Fennell T.R., Silinski M.R., Watson S.L., Licause J., Robinson V.G., Sparrow B. (2021). Toxicokinetic evaluation of the common indoor air pollutant, α-pinene, and its potential reactive metabolite, α-pinene oxide, following inhalation exposure in rodents. Toxicol. Appl. Pharmacol..

[27] Wang F., Li C., Liu W., Jin Y. (2014). Potential mechanisms of neurobehavioral disturbances in mice caused by sub-chronic exposure to low-dose VOCs. Inhal. Toxicol..

[28] Skulberg K., Nyrud A., Goffeng L., Wisthaler A. (2019). Health and exposure to VOCs from pinewood in indoor environments. Front. Built Environ..

[29] He W., Rahman M.H., Bajgai J., Abdul-Nasir S., Mo C., Ma H., Goh S.H., Bomi K., Jung H., Kim C.-S. (2024). Hydrogen Gas Inhalation Alleviates Airway Inflammation and Oxidative Stress on Ovalbumin-Induced Asthmatic BALB/c Mouse Model. Antioxidants.

[30] Ma H., Kim K.-H., Eom C.-D., Rahman M.H., Bajgai J., Abdul-Nasir S., Mo C., Hwang W.-J., Goh S.H., Kim B. (2025). Impact of Short- and Long-Term Exposure to Engineered Wood (Plywood and Particle Board) on Immune and Oxidative Biomarkers: A C57BL/6 Mouse Model Study. Polymers.

[31] Glenn A., Armstrong C.E. (2019). Physiology of red and white blood cells. Anaesth. Intensive Care Med..

[32] Hong Y., Boiti A., Vallone D., Foulkes N.S. (2024). Reactive oxygen species signaling and oxidative stress: Transcriptional regulation and evolution. Antioxidants.

[33] Kany S., Vollrath J.T., Relja B. (2019). Cytokines in inflammatory disease. Int. J. Mol. Sci..

[34] Schroder K., Hertzog P.J., Ravasi T., Hume D.A. (2004). Interferon-γ: An overview of signals, mechanisms and functions. J. Leukoc. Biol..

[35] Lopez-Castejon G., Brough D. (2011). Understanding the mechanism of IL-1β secretion. Cytokine Growth Factor Rev..

[36] Tanaka T., Narazaki M., Kishimoto T. (2014). IL-6 in inflammation, immunity, and disease. Cold Spring Harb. Perspect. Biol..

[37] Kalliolias G.D., Ivashkiv L.B. (2016). TNF biology, pathogenic mechanisms and emerging therapeutic strategies. Nat. Rev. Rheumatol..

[38] Saraiva M., O’Garra A. (2010). The regulation of IL-10 production by immune cells. Nat. Rev. Immunol..

[39] Lim J., Moon G., Lee M., Kang J., Won M., Ahn E., Jeon J. (2020). Korea’s Forest Resources.

[40] Farjon A. (2010). A Handbook of the World’s Conifers: Revised and Updated Edition.

[41] Frezza C., De Vita D., Sciubba F., Toniolo C., Tomassini L., Nicoletti M., Franceschin M., Guiso M., Bianco A., Serafini M. (2022). There is not only *Cupressus sempervirens* L.: A review on the phytochemistry and bioactivities of the other *Cupressus* L. species. Appl. Sci..

[42] Absollhi M. (2014). Encyclopedia of Toxicology.

[43] Franck U., Weller A., Röder S.W., Herberth G., Junge K.M., Kohajda T., von Bergen M., Rolle-Kampczyk U., Diez U., Borte M. (2014). Prenatal VOC exposure and redecoration are related to wheezing in early infancy. Environ. Int..

[44] Mubbunu L., Bowa K., Petrenko V., Silitongo M. (2018). Correlation of internal organ weights with body weight and body height in normal adult Zambians: A case study of Ndola Teaching Hospital. Anat. Res. Int..

[45] Lappas C.M., Lappas N.T. (2012). D-Limonene modulates T lymphocyte activity and viability. Cell. Immunol..

[46] Ogbodo J.O., Arazu A.V., Iguh T.C., Onwodi N.J., Ezike T.C. (2022). Volatile organic compounds: A proinflammatory activator in autoimmune diseases. Front. Immunol..

[47] Ghelli F., Bellisario V., Squillacioti G., Grignani E., Garzaro G., Buglisi M., Bergamaschi E., Bono R. (2021). Oxidative stress induction in woodworkers occupationally exposed to wood dust and formaldehyde. J. Occup. Med. Toxicol..

[48] McGill M.R. (2016). The past and present of serum aminotransferases and the future of liver injury biomarkers. EXCLI J..

[49] O’Donnell M.P., Burne M., Daniels F., Rabb H. (2002). Utility and limitations of serum creatinine as a measure of renal function in experimental renal ischemia-reperfusion injury. Transplantation.

[50] Krinsley J., Schultz M., Spronk P., Harmsen R., van Braam Houckgeest F., Van der Sluijs J., Mélot C., Preiser J.C. (2011). Mild hypoglycemia is independently associated with increased mortality in the critically ill. Crit. Care.

[51] Muthusamy S., Peng C., Ng J.C. (2016). Effects of binary mixtures of benzo[a]pyrene, arsenic, cadmium, and lead on oxidative stress and toxicity in HepG2 cells. Chemosphere.

[52] Klein R., Nagy O., Tóthová C., Chovanová F. (2020). Clinical and diagnostic significance of lactate dehydrogenase and its isoenzymes in animals. Vet. Med. Int..

[53] Miekisch W., Schubert J.K., Noeldge-Schomburg G.F. (2004). Diagnostic potential of breath analysis—Focus on volatile organic compounds. Clin. Chim. Acta.

[54] Laverty H.G., Antoine D.J., Benson C., Chaponda M., Williams D., Park B.K. (2010). The potential of cytokines as safety biomarkers for drug-induced liver injury. Eur. J. Clin. Pharmacol..

[55] Neuman M.G., Cohen L.B., Nanau R.M. (2014). Biomarkers in nonalcoholic fatty liver disease. Can. J. Gastroenterol. Hepatol..

[56] Aboderin F.I., Oduola T., Davison G.M., Oguntibeju O.O. (2023). A review of the relationship between the immune response, inflammation, oxidative stress, and the pathogenesis of sickle cell anaemia. Biomedicines.

[57] Morawska I., Kurkowska S., Bębnowska D., Hrynkiewicz R., Becht R., Michalski A., Piwowarska-Bilska H., Birkenfeld B., Załuska-Ogryzek K., Grywalska E. (2021). The epidemiology and clinical presentations of atopic diseases in selective IgA deficiency. J. Clin. Med..

[58] Kim D.-S., Lee H.-J., Jeon Y.-D., Han Y.-H., Kee J.-Y., Kim H.-J., Shin H.-J., Kang J., Lee B.S., Kim S.-H. (2015). Alpha-pinene exhibits anti-inflammatory activity through the suppression of MAPKs and the NF-κB pathway in mouse peritoneal macrophages. Am. J. Chin. Med..

[59] Yang J., Choi W.-S., Kim J.-W., Lee S.-S., Park M.-J. (2019). Anti-inflammatory effect of essential oils extracted from wood of four coniferous tree species. J. Korean Wood Sci. Technol..

[60] Leite P.M., Amorim J.M., Castilho R.O. (2022). Immunomodulatory role of terpenoids and phytosteroids. Plants and Phytomolecules for Immunomodulation: Recent Trends and Advances.

[61] Hamza T., Barnett J.B., Li B. (2010). Interleukin 12, a key immunoregulatory cytokine in infection applications. Int. J. Mol. Sci..

[62] Chaudhary S., Siddiqui M., Athar M., Alam M.S. (2012). D-Limonene modulates inflammation, oxidative stress and Ras-ERK pathway to inhibit murine skin tumorigenesis. Hum. Exp. Toxicol..

