# Supplementary material for: Immune-Redox Biomarker Responses to Short- and Long-Term Exposure to Naturally Emitted Compounds from Korean Red Pine (Pinus densiflora) and Japanese Cypress (Chamaecyparis obtusa): In Vivo Study
Source: Toxics. 2025 Jul 31;13(8):650. doi: 10.3390/toxics13080650 (PMC12389784; doi:10.3390/toxics13080650)
Supplement: Supplementary file 1 [file toxics-13-00650-s001.zip › toxics-3711632-supplementary.pdf]

Supplemental Information

Table S1. Total Number of VOCs and Area Percentage Change of Positive and Negative VOC Release Areas from Korea Red Pine (*Pinus densiflora*) and Japanese Cypress (*Chamaecyparis obtusa*) over 30 - 180 Days.

**Time**	**Korea Red Pine**			**Japanese Cypress**		
	**No.**	**Positive (Area%)**	**Negative (Area%)**	**No.**	**Positive (Area%)**	**Negative (Area%)**
**30 Days**	36	82.40	5.30	65	74.10	2.00
**60 Days**	46	82.20	8.70	71	73.60	2.20
**120 Days**	50	79.73	6.32	71	74.04	1.80
**180 Days**	58	73.4	13.10	80	69.00	4.00

Table S2. Main Positive VOCs and Trend Analysis for Korea Red Pine (*Pinus densiflora*) over 30 - 180 Days.

Korea Red Pine (*Pinus densiflora*)					
**No.**	**VOC Name**	**30 Days (Area%)**	**60 Days (Area%)**	**120 Days (Area%)**	**180 Days (Area%)**
1	alpha-Pinene	26.00	22.80	27.55	23.00
2	Longifolene	23.40	24.40	22.95	22.90
3	alpha-Terpineol	9.10	9.40	4.76	5.70
4	Benzaldehyde	5.50	4.80	2.89	4.00
5	Acetic acid	4.60	4.70	2.78	3.40
6	D-Limonene	3.80	3.50	3.80	3.30
7	Camphene	1.80	1.70	1.93	1.90
8	o-Cymene	1.50	1.30	1.63	2.00
9	alpha-Longipinene	1.30	1.30	1.65	1.30
10	beta-Pinene	0.60	0.60	2.96	0.40
11	Sabinene	1.40	1.10	2.53	-
12	Cembrene	-	3.20	2.36	2.00
13	delta-Amorphene	1.00	1.00	-	-
14	Terpinen-4-ol	0.50	0.60	-	0.30
15	alpha-Terpilene	0.40	-	0.40	-
16	Fenchol	0.80	-	0.43	-
17	alpha-Phellandrene	-	-	0.72	0.70
18	Thymol methyl ether	0.70	-	-	-
19	alpha-Campholenal	-	1.00	-	-
20	(+)-Cyclosativene	-	0.80	-	-
21	delta-Cadinene	-	-	0.42	-
22	Longicyclene	-	-	-	1.30
23	(+)-Sativene	-	-	-	1.20
	**Total**	**82.40**	**82.2**	**79.73**	**73.4**

Table S3. Main Negative VOCs and Trend Analysis for Korea Red Pine (*Pinus densiflora*) over 30 - 180 Days.

Korea Red Pine (*Pinus densiflora*)					
**No.**	**VOC Name**	**30 Days (Area%)**	**60 Days (Area%)**	**120 Days (Area%)**	**180 Days (Area%)**
1	Methanol	2.30	2.30	0.78	1.00
2	Hexanal	0.80	1.30	2.23	5.10
3	(+)-Sativene	1.00	1.00	1.44	-
4	o-Isopropenyltoluene	0.50	-	0.58	0.90
5	Acetone	0.70	0.70	-	0.90
6	Furfural	-	1.300	0.54	0.80
7	Hexanoic acid	-	-	0.75	1.20
8	1-Pentanol	-	-	-	0.50
9	Linalol	-	0.70	-	0.50
10	Nonanal	-	-	-	1.00
11	Styrene	-	0.70	-	-
12	2-Methyl-1-phenylpropene	-	0.70	-	-
13	beta-Phellandrene	-	-	-	1.20
	**Total**	**5.30**	**8.70**	**6.32**	**13.10**

Table S4. Main Positive VOCs and Trend Analysis for Japanese Cypress (*Chamaecyparis obtusa*) over 30 - 180 Days.

Japanese Cypress (*Chamaecyparis obtusa*)					
**No.**	**VOC Name**	**30 Days (Area%)**	**60 Days (Area%)**	**120 Days (Area%)**	**180 Days (Area%)**
1	alpha-Pinene	13.70	12.40	13.88	12.00
2	alpha-Cadinol	10.70	10.10	9.73	9.60
3	alpha-Muurolene	6.20	5.90	5.42	5.60
4	tau-Cadinol	8.10	-	9.86	6.60
5	alpha-Terpineol	3.90	3.80	3.22	3.20
6	D-Limonene	2.10	2.00	2.79	2.50
7	Acetic acid	1.60	1.50	1.22	1.10
8	alpha-Terpinyl acetate	1.10	1.30	1.00	1.00
9	(+)-beta-Selinene	1.10	1.10	0.99	0.90
10	cis-Calamene	1.10	1.40	1.05	1.20
11	Terpinen-4-ol	0.90	0.90	0.9	0.90
12	gamma-Muurolene	4.90	4.70	4.41	-
13	alpha-Amorphene	2.40	2.00	1.82	-
14	beta-Cadinene	-	12.80	1.86	2.20
15	delta-Cadinene	-	-	14.52	14.40
16	endo-Borneol	1.10	1.00	-	-
17	beta-Calacorene	-	2.50	-	2.20
18	delta-Amorphene	13.70	-	-	-
19	tau-Muurolol	-	10.20	-	-
20	alpha-Calacorene	1.50	-	-	-
21	Borneol	-	-	0.79	-
22	gamma-Cadinene	-	-	-	4.70
23	(+)-beta-Thujene	-	-	-	0.90
24	Camphene	-	-	0.58	-
	**Total**	**74.1**	**73.6**	**74.04**	**69.00**

Table S5. Main Negative VOCs and Trend Analysis for Japanese Cypress (*Chamaecyparis obtusa*) over 30 - 180 Days.

Japanese Cypress (*Chamaecyparis obtusa*)					
**No.**	**VOC Name**	**30 Days (Area%)**	**60 Days (Area%)**	**120 Days (Area%)**	**180 Days (Area%)**
1	Methanol	0.80	0.80	0.50	0.50
2	Acetone	0.30	0.50	0.46	0.90
3	Furfural	0.30	0.20	0.12	0.10
4	o-Isopropenyltoluene	0.60	-	0.72	0.90
5	Verbenone	-	-	-	0.50
6	Isopropyl Alcohol	-	-	-	0.20
7	2-Acetyltoluene	-	-	-	0.20
8	Hexanal	-	-	-	0.10
9	Toluene	-	-	-	0.60
10	Styrene	-	0.70	-	-
	**Total**	**2.00**	**2.20**	**1.80**	**4.00**

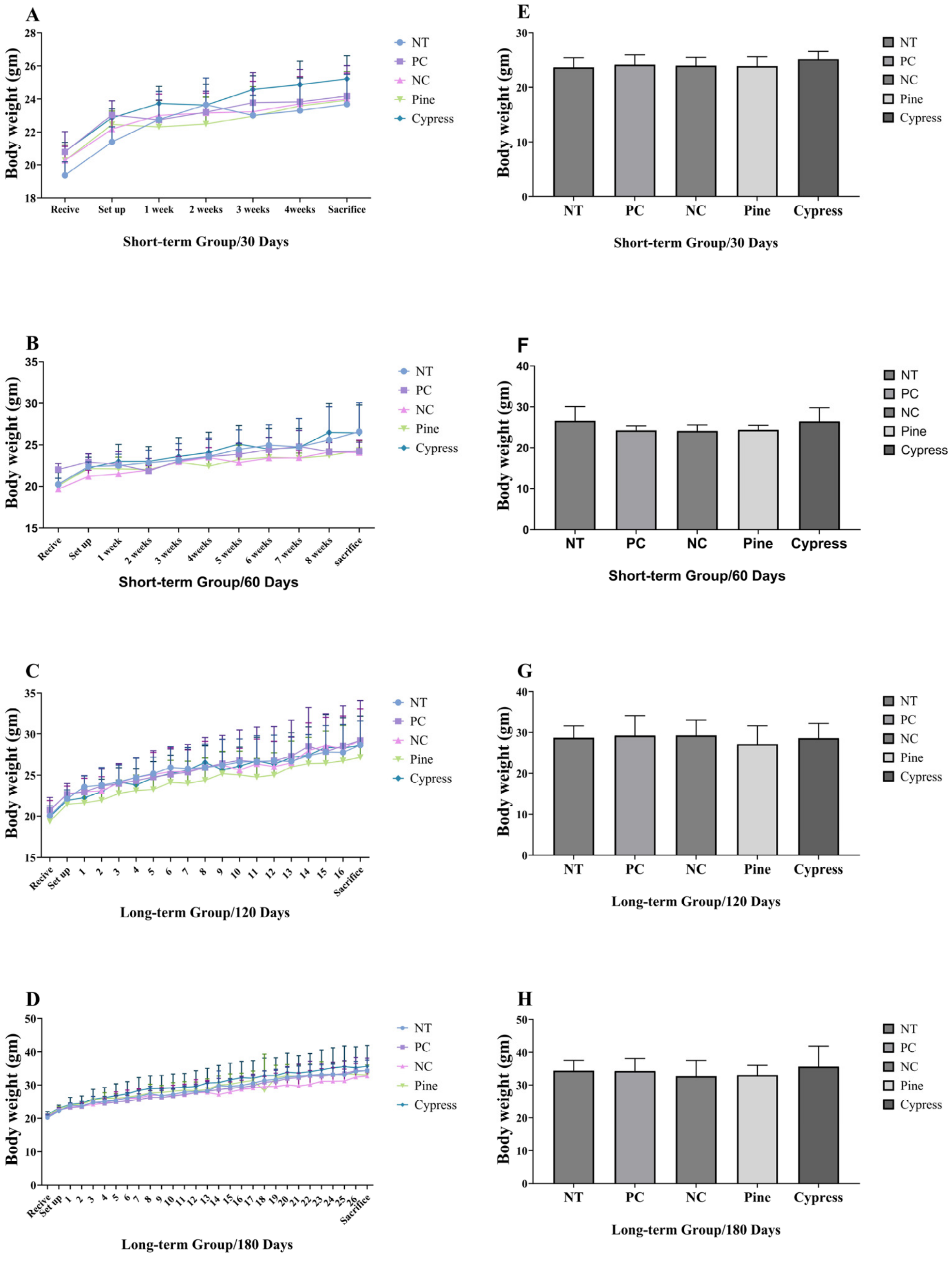

Figure S1. Short-term 30-60 days and Long-term 120-180days effects on body weight of VOCs released from wood samples in the mice cages. A-D: the weekly body weight measurement data of 30-180days; E-H: Body weight measurement data of 30-180days before sacrifice. Data is shown as mean ± SD for n=4 mice for short-term groups and n=8 mice for long-term groups.

Abbreviations: NT, non-treated control; PC: phytoncide exposure group; NC: formaldehyde exposure group; Pine: Korea Red Pine wood exposure group; Cypress: Japanese Cypress wood exposure group. These abbreviations apply to all the following figures.

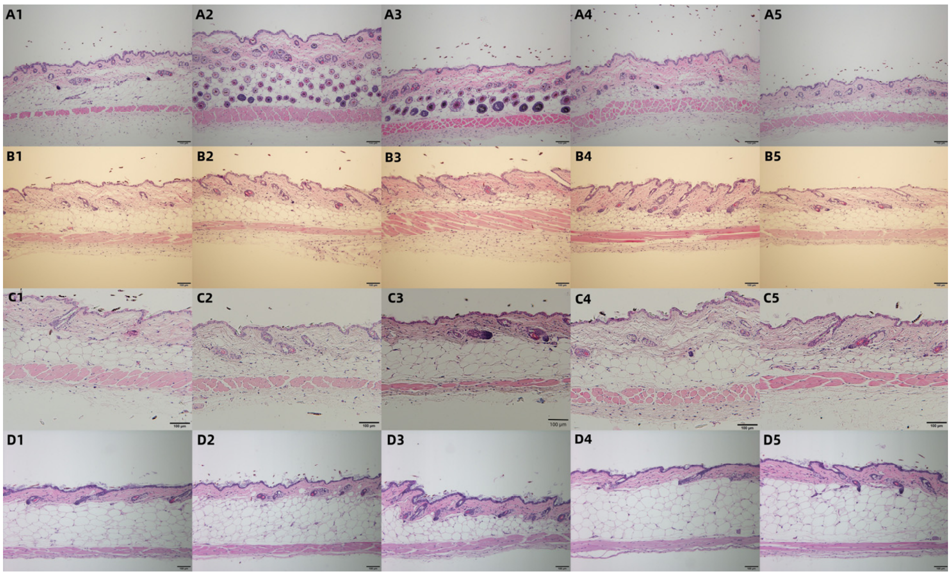

Figure S2. The H&E Staining of Skin. A: 30days; B: 60days; C: 120days; D:180days; 1: NT Group; 2: PC Group; 3: NC group; 4: EG1 group; 5: EG2 group; Hematoxylin and eosin (H&E) staining, bar=50μm; bar=100μm; bar=200μm.

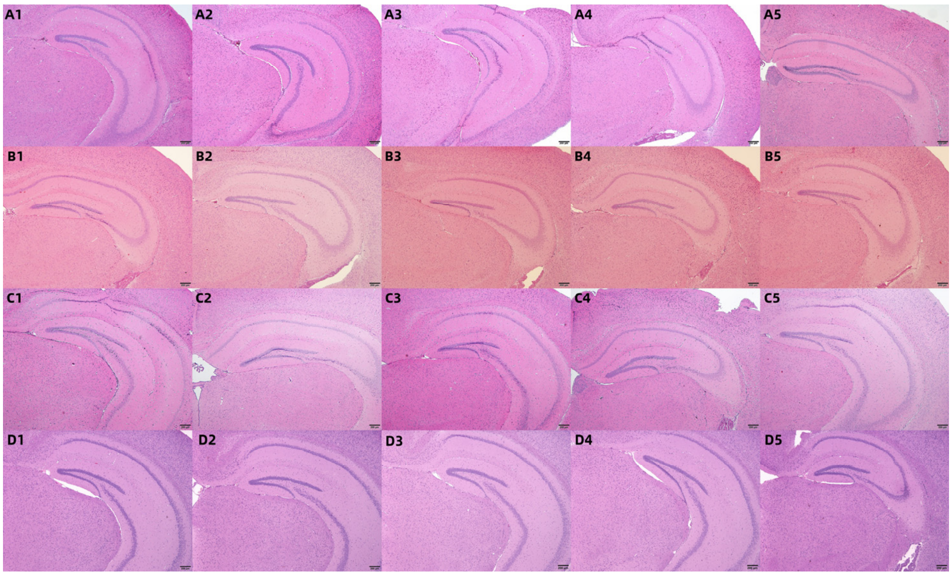

Figure S3. The H&E Staining of Brian. A: 30days; B: 60days; C: 120days; D:180days; 1: NT Group; 2: PC Group; 3: NC group; 4: EG1 group; 5: EG2 group; Hematoxylin and eosin (H&E) staining, bar=50μm; bar=100μm; bar=200μm.

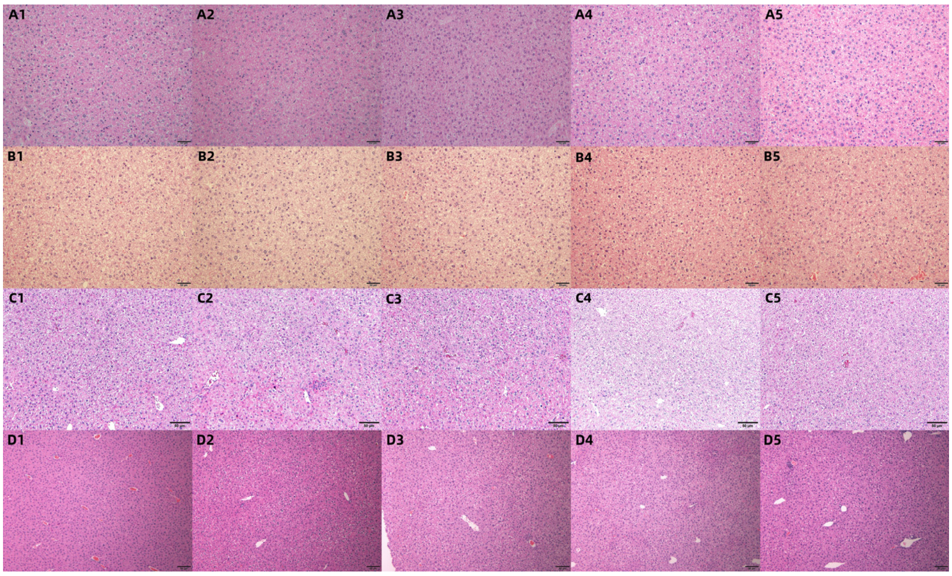

Figure S4. The H&E Staining of Liver. A: 30days; B: 60days; C: 120days; D:180days; 1: NT Group; 2: PC Group; 3: NC group; 4: EG1 group; 5: EG2 group; Hematoxylin and eosin (H&E) staining, bar=50μm; bar=100μm; bar=200μm.

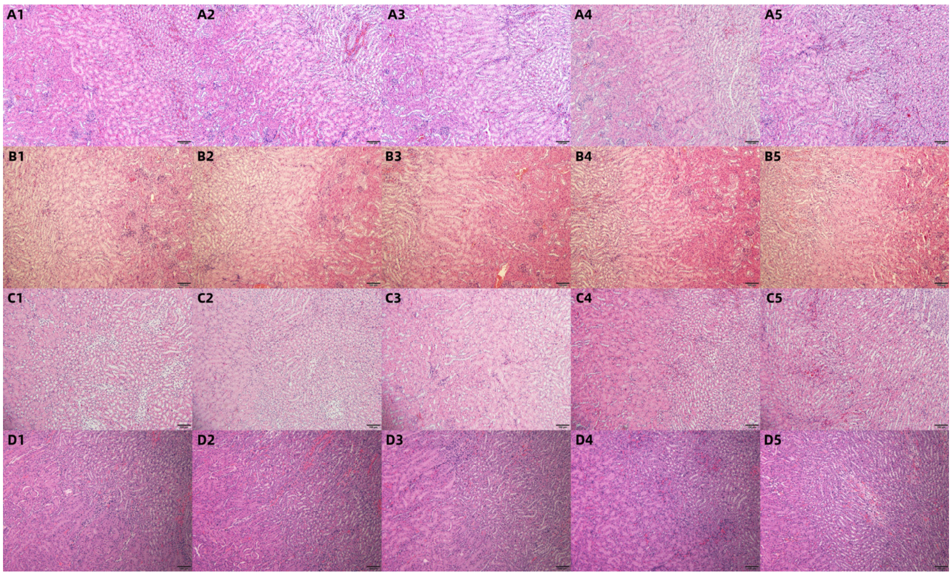

Figure S5. The H&E Staining of Kidney. A: 30days; B: 60days; C: 120days; D:180days; 1: NT Group; 2: PC Group; 3: NC group; 4: EG1 group; 5: EG2 group; Hematoxylin and eosin (H&E) staining, bar=50μm; bar=100μm; bar=200μm.

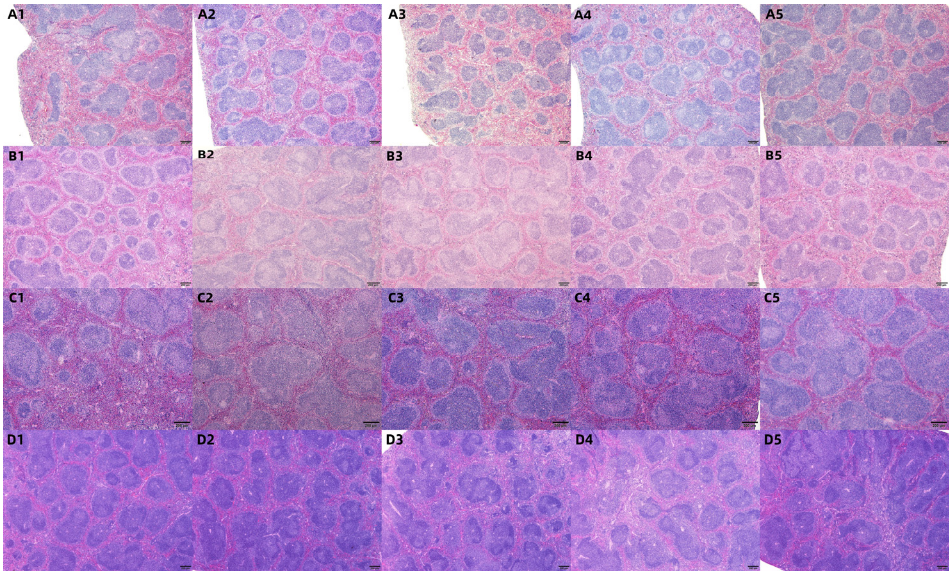

Figure S6. The H&E Staining of Spleen. A: 30days; B: 60days; C: 120days; D:180days; 1: NT Group; 2: PC Group; 3: NC group; 4: EG1 group; 5: EG2 group; Hematoxylin and eosin (H&E) staining, bar=50μm; bar=100μm; bar=200μm.
